# Bayesian Models to Generate Small Area Estimates of Population Health: Tutorial for Using Rate Stabilizing Tools and Their Output

**DOI:** 10.2196/83498

**Published:** 2026-01-30

**Authors:** David DeLara, Ryan Zomorrodi, Harrison Quick, Joshua Tootoo, Ruiyang Li, Justan Baker, Jihyeon Kwon, Michele Casper, Adam Vaughan

**Affiliations:** 1 Division for Heart Disease and Stroke Prevention, National Center for Chronic Disease Prevention and Health Promotion, Centers for Disease Control and Prevention, 4770 Buford Highway, Atlanta, GA, United States, 1 770-488-8976; 2 Children's Environmental Health Initiative, University of Illinois Chicago, Chicago, IL, United States; 3 Division of Biostatistics and Health Data Science, University of Minnesota, Minneapolis, MN, United States; 4 Diabetes and Cardiovascular Health Program, Rhode Island Department of Health, Providence, RI, United States; 5 Department of Epidemiology and Biostatistics, Drexel University, Philadelphia, PA, United States

**Keywords:** small area estimates, spatial analysis, software, geographic information system, GIS, spatiotemporal models, R, Bayesian statistics

## Abstract

The demand for high-quality population health data at the local level calls for expanded tools for those working to enhance the health of communities across the country to easily calculate small area estimates. Statistical models that generate small area estimates often use Bayesian estimation techniques, which are computationally complex and not readily accessible to most public health professionals. We developed 2 tools to facilitate small area estimation. For ArcGIS Pro users, we developed the Rate Stabilizing Toolbox ArcGIS plugin (RSTbx), and for R users, we developed the Rate Stabilizing Tool R package (RSTr). In this tutorial, we demonstrate how to use these tools to calculate small area estimates and evaluate their reliability. We also demonstrate 3 key benefits from using either of these tools: (1) decreased number of geographic units with suppressed estimates, (2) flexibility to set the threshold for statistical reliability, and (3) credible intervals that can be used to identify statistically significant differences between geographic units. Additionally, both tools offer built-in age-standardization capabilities. We created census tract–level maps from North Carolina mortality data and Rhode Island hospitalization data to showcase the benefits of generating small area estimates with these tools. Rate Stabilizing Toolbox and Rate Stabilizing Tool for R are powerful tools that can be used to meet the demand for high-quality local-level data to inform public health programs and tailor health promotion activities to the needs of communities across the country.

## Introduction

**Figure 1.**
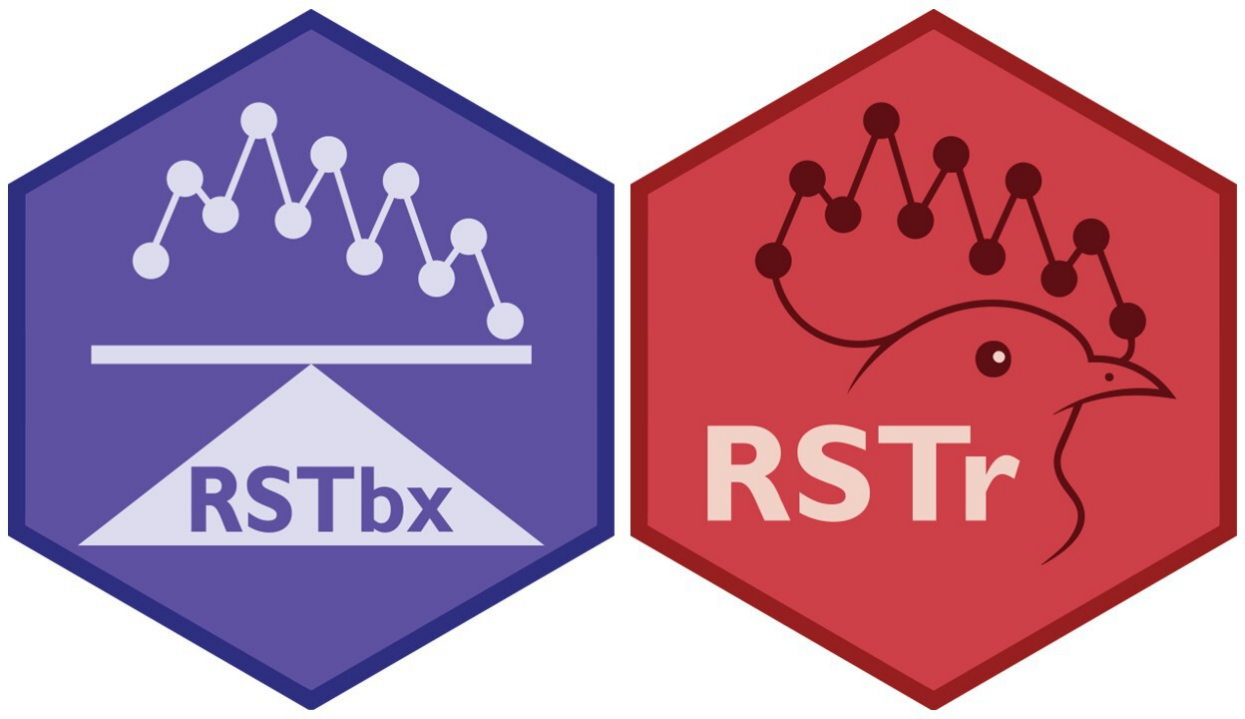
Icons for Rate Stabilizing Toolbox (RSTbx; left) and Rate Stabilizing Tool for R (RSTr; right).

The demand for high-quality population health data at the local level calls for expanded tools that public health professionals and others can use to easily calculate robust small area estimates [1-3]. A key challenge for calculating robust small area estimates occurs in regions with small population sizes or few numbers of events; these small numbers introduce statistical uncertainty, leading to challenges in the meaningful interpretation of results [4]. Statistical methods that appropriately address these challenges are often computationally complex and may not be readily accessible to those working to enhance the health of communities across the country. In response to this need, we implemented established statistical models into 2 distinct tools: an ArcGIS toolbox (the Rate Stabilizing Toolbox [RSTbx]) and an R package (the Rate Stabilizing Tool for R [RSTr]) (Figure 1). These tools enable users to input local-level data and calculate robust small area estimates. This tutorial provides step-by-step instructions for how to use each tool along with a demonstration of the benefits derived from using these tools.

Based on Tobler's First Law of Geography [5], which asserts that closer places are more alike than further places, spatial statistical models improve estimate precision by leveraging the data’s underlying spatial structure (ie, spatial smoothing). Among these existing models, RSTbx and RSTr are based on the conditional autoregressive (CAR) model developed by Besag, York, and Mollié (BYM) [6], which is used extensively in spatial epidemiology and disease mapping [7]. Outcomes as varied as excess cardiovascular disease death rates in the United States during the COVID-19 pandemic [8], tuberculosis relative risk in Indonesia [9], and under-nutrition among under-five children in Ethiopia [10] have recently been investigated with methodology based on the BYM model. The popularity of the BYM model is further enhanced due to its extensions into spatiotemporal and multivariate settings through the MCAR of Gelfand and Vounatsou [11] and the MSTCAR of Quick et al [12]. Recent developments in BYM models involve strategies to quantify model informativeness and avoid oversmoothing [13,14] and outlining of standards for estimate reliability [15].

The RSTbx and RSTr both offer key benefits for calculating and mapping small area estimates of population health. First, by leveraging spatial and other dependencies in the data, the estimates produced by the RSTbx and RSTr are more precise than those based solely on the observed data. As such, the estimates produced by these tools will be more reliable, resulting in fewer estimates being suppressed, thereby permitting the documentation of geographic patterns with a more comprehensive geographic coverage. Second, both tools allow users to relax the threshold for reliability based on measures of uncertainty (eg, basing thresholds for reliability on the 80% credible intervals [CIs] rather than the standard 95% CIs). Third, these measures of uncertainty can be used to assess statistically significant differences between estimates for geographic units and other domains.

We have divided this tutorial into 3 sections. We begin with an overview of each tool, including how to use the tool, the input and output datasets, and their modeling capabilities. Then, we demonstrate the benefits of using the tools by mapping small area estimates of mortality using RSTbx and hospitalization rates using RSTr. Finally, we review the tools’ strengths and limitations and provide a table comparing their major features.

## RSTbx: An ArcGIS Toolbox

### Overview

RSTbx is a Python-based set of tools designed for Environmental Systems Research Institute’s ArcGIS Pro software [16]. Users can input their own local-level data and calculate local-level estimates using Bayesian spatial smoothing methods. RSTbx uses a BYM model framework that smooths across space using data from adjacent geographic units [6]. RSTbx also includes options for data processing, age-standardization, and the generation of CIs. RSTbx, with detailed instructions, is available for download on GitHub [16].

RSTbx is an upgrade to the original Rate Stabilizing Tool (RST) that was created in 2019 [17]. Enhancements include the following:

- A CAR model implementation based on the BYM model to replace the Poisson-gamma empirical Bayes method used in the original RST [7,8].
- The user interface has been revamped for greater ease of use.
- Users can import custom features; they are no longer limited to 2010 US Census geographies.
- Users can import their own population tables, whereas the original RST allowed only population data from the 2010 Census of the Population.
- Age-standardization to 10-year age groups is now possible using either the 2000 US Standard Population or the 2010 US Standard population [18].
- Population tables from the decennial US Census and American Community Survey (ACS; by census tract or county) can now be downloaded directly into an ArcGIS Pro project along with US Census TIGER and cartographic geographic boundaries [19].

RSTbx includes 3 tools: the Census Data Retriever (CDR), the Individual Data Processor (IDP), and the RST. The CDR generates population tables at the county or census tract level using data from the US Census’ Decennial Census or the ACS. It can also be used to download TIGER or cartographic boundaries. The IDP aggregates individual-level event data by calculating the number of events within each geographic unit and joining the aggregate counts to the provided population table. The IDP can aggregate individual-level event data by age group to produce aggregate data for each geographic unit. The IDP also performs several data validation checks (eg, the IDP will check for null data, incorrect datatypes, or duplicate geographic units within population data). Finally, the RST runs a BYM model and generates small area estimates.

### Installing RSTbx

To install RSTbx, users first download the latest release from the GitHub repository as a zip file [16]. After unzipping this file, users should open an ArcGIS Project, navigate to the Catalog Pane, right-click on Toolboxes, click on Add Toolbox, and find the rate_stabilizing_toolbox.pyt file within the unzipped RSTbx folder. After completing these steps, RSTbx is ready to use.

### Input Data Requirements

RSTbx requires a minimum of 3 sets of data: event data, population data, and a boundary file. First, users enter event data for the health outcome of interest. RSTbx is designed to accommodate event data at either the individual or group level. For individual-level event data, RSTbx requires a unique identifier for each geographic unit (eg, GEOID). The age of each individual is also required to calculate age-standardized rates. For group-level event data, RSTbx requires a unique identifier for each geographic unit, the number of events in each geographic unit (ie, the numerator for the health outcome of interest), and the age group. The age group is optional and only required if users want to generate age-standardized rates. If the age group is included, the groups are restricted to: “0-4,” “5-14,” “15-24,” “25-34,” “35-44,” “45-54,” “55-64,” “65-74,” “75-84,” “85up.” For both the event and population data, the typical use case is single-year data, but data can be analyzed along any temporal aggregation.

Second, users must provide a table with the population data for each geographic unit or geographic unit–age group combination. Users can either supply their own population-level data or access the CDR within RSTbx to retrieve population data for census geographies. Users with noncensus geographies must provide their own population-level data. Importantly, RSTbx assumes that data for each geographic unit are a census (ie, data represent the entire population).

Finally, users enter a boundary file for the geographic area of interest. RSTbx supports most major file types through ArcGIS Pro, including but not limited to Geodatabase features, GeoPackage features, GeoParquets, and shapefiles. Each geographic unit must have at least 1 neighbor. If users are not using their own geographic boundary files, the CDR can be used to download TIGER or cartographic boundaries from the US Census Bureau.

### Generating Small Area Estimates With RSTbx

**Figure 2.**
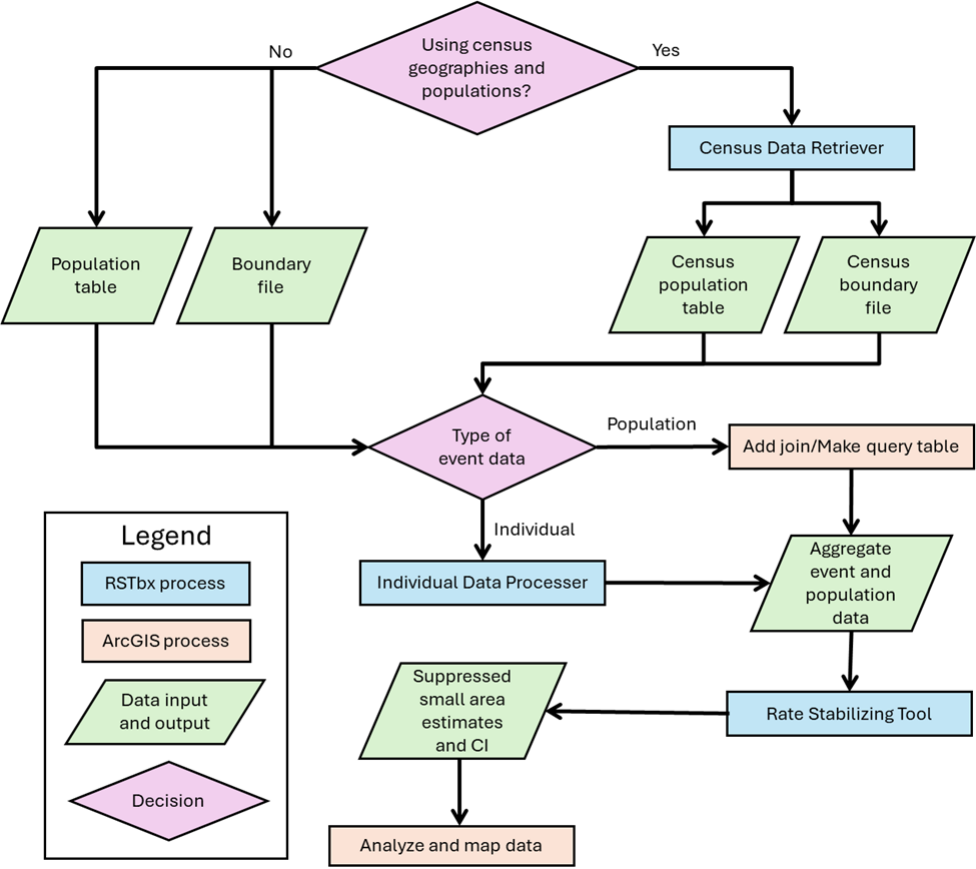
Flowchart for generating small area estimates using the Rate Stabilizing Toolbox (RSTbx).

Figure 2 outlines the steps involved in using RSTbx to calculate local-level measures of population health. The first steps include determining the geographic unit of analysis and obtaining population data and a boundary file for the chosen geographic unit. If the event data (eg, health data) are at the individual level, they must be aggregated to the geographic unit and joined to the population table with the IDP; population-level event data may be directly joined to the population table. The next step entails choosing the desired threshold for reliability. Finally, if conducting age-standardization, it is necessary to choose the age groups and a standard population year to be used in the age-standardization process. Detailed directions are provided within RSTbx and within the GitHub repository [16].

### Output

After running the models, RSTbx generates samples from the posterior distribution for each geographic unit, from which an output table is created. The table includes spatially smoothed estimates (ie, the posterior medians), lower and upper bounds of the user-defined CIs, and the level of reliability (eg, 95%, 90%, 75%) for each geographic unit. If age-standardization is requested, results will be produced for the age-standardized age range and all composite age groups within the data.

## RSTr R Package

### Overview

RSTr is an R package using Rcpp and RcppArmadillo [20-23] that generates small area estimates of health outcomes using CAR models [24]. Users may choose from one of many BYM-based models to generate small area estimates, including the univariate CAR, multivariate CAR (MCAR), and multivariate spatiotemporal CAR (MSTCAR). RSTr’s CAR model smooths across space using data from neighboring geographic units [6,13]. The MCAR expands upon the CAR model by smoothing across both geographic units and domains (eg, sociodemographic groups) [11]. The MSTCAR further expands the MCAR model by smoothing across geographic units, domains, and time [12]. Statistical and technical details of these models are available in the package documentation [24].

### Package Setup

RSTr uses the R statistical software [25] and can be installed from the Comprehensive R Archive Network (CRAN) [24].

### Input Data Requirements

RSTr requires 3 pieces of input data: event counts, population data, and the adjacency structure of the geographic units. RSTr uses aggregate, rather than individual-level, data. Requirements for the structure of these inputs are described in detail in the package documentation [24]. Briefly, datasets for the event and population data for each geographic unit should be combined into an R list object. Requirements for the data structure differ based on the selected model. For example, the CAR model uses vectors, the MCAR model uses matrices, and the MSTCAR model uses 3-dimensional arrays. Importantly, RSTr assumes that data for each geographic unit represent a census (ie, data represent the entire population). For both event and population data, the typical use case is single-year data, but data can be analyzed along any temporal aggregation. For the MSTCAR model, any temporal aggregation can be used, but all time periods must be an equal distance apart. Users must supply their own population count data, but can easily acquire data through the use of the tidycensus package for downloading 1-year and 5-year ACS estimates, along with 10-year decennial census data [26]. Finally, RSTr’s models will impute data for estimates censored due to privacy reasons. For additional information about the data setup process, refer to the vignette titled “01: Understanding and Preparing Your Event Data.”

Adjacency information tells RSTr the neighbors of each geographic unit. Each geographic unit must have at least 1 neighbor. These adjacency data should be formatted in a structure the same as that generated by the poly2nb() function from the spdep package [27,28]. RSTr supports any filetype supported by GDAL through the use of the sf package [29,30], which has drivers for nearly every geospatial format.

### Generating Small Area Estimates With RSTr

**Figure 3.**
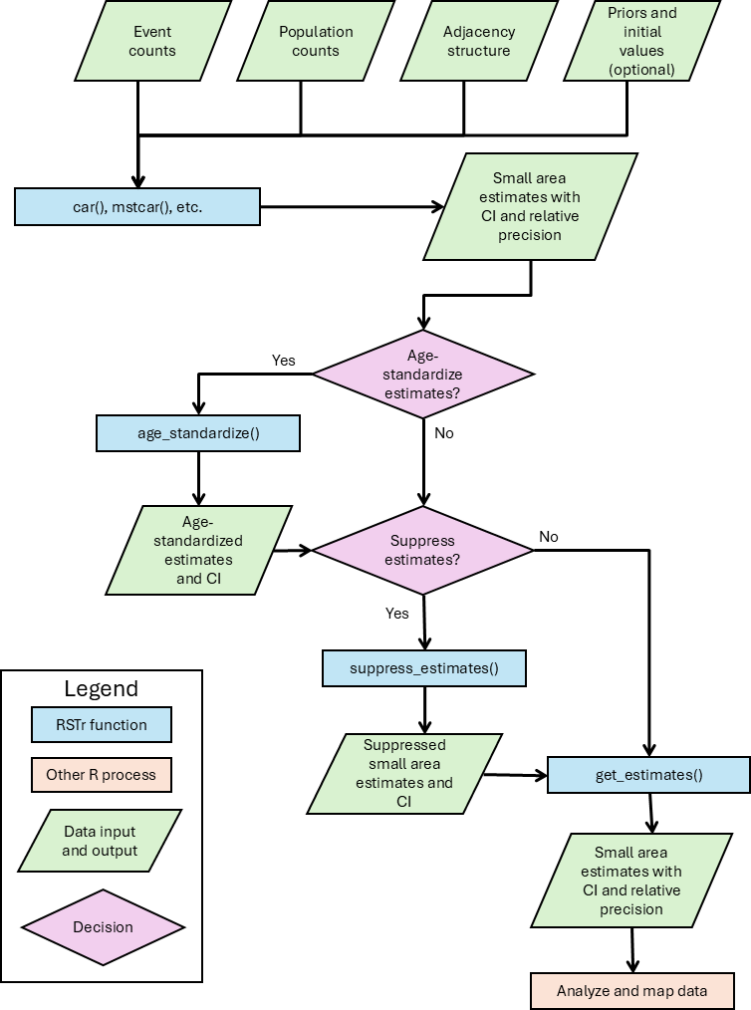
Flowchart for generating small area estimates using the Rate Stabilizing Tool for R (RSTr).

Figure 3 provides an outline of how to run models with RSTr. When running the model, users specify, at a minimum, the event data, population data, and adjacency structure. However, users may also specify the distribution of the event data (either Poisson or binomial as appropriate), initial values, priors, and other parameters to tune the model. Historically, birth and death data have been assumed to arise from Poisson processes [31], thus prompting the use of Poisson distributions to model birth [14,15] and death data [32,33]. However, because the Poisson distribution can be used to approximate the binomial distribution—and because a binomial might be more appropriate for other types of data—RSTr defaults to using a binomial distribution.

The decision of which model to select depends on the nature of the underlying data. In particular, the UCAR model is appropriate for a single year of data and a single demographic group, while the MCAR model is appropriate for a single year and multiple demographic groups (ie, multiple age groups to generate age-standardized estimates) and the MSTCAR model is appropriate for data with both multiple years of data and multiple groups.

### Output

Typical of Bayesian models, the RSTr models generate output in the form of samples. These samples are then used to generate small area estimates and CIs. Samples are included as a standalone output; estimates based on those samples, along with their corresponding CIs and relative precisions, are included as part of a larger model object from which these elements can be extracted.

Estimates are defined as the median of these samples and are extracted using the get_estimates() function on the RSTr model object. The output of get_estimates() is by default a long table containing medians (ie, estimates) for each region, group, and time period included in the model. The get_estimates() function also returns the credible interval, event and population counts, and relative precision, defined by Quick et al [13] as the ratio of the posterior median and the width of the user-specified CI, for each estimate. Relative precisions greater than 1 correspond to “reliable” estimates, per the framework of Quick et al [13].

Users may specify thresholds for reliability from 0 to 1 to define the CI used for relative precision calculations. At the default level of 0.95, the CI is defined as the 2.5 and 97.5 percentiles of the samples. This function may be especially beneficial when data are limited and few, if any, estimates would be deemed reliable at the traditional 0.95 level. For example, presenting maps of the estimates that are reliable at the 0.80 level will allow more estimates to be displayed while also acknowledging that a relaxed standard of reliability is being used. This functionality is demonstrated below.

## Benefits of Mapping Small Area Estimates Generated by RSTbx and RSTr

### Overview

There are many benefits to mapping small area estimates of population health generated by the RSTbx and RSTr. In this section, we demonstrate 3 key benefits: (1) decreased number of geographic units with suppressed estimates, (2) flexibility to set a threshold for reliability, and (3) CIs that can be used to identify statistically significant differences between geographic units.

### Datasets Used to Demonstrate the Benefits of RSTbx and RSTr

#### RSTbx: North Carolina Mortality Data

To demonstrate the benefits of the RSTbx, we used North Carolina mortality data at the census tract level. Specifically, we examined heart disease deaths among adults aged 35 to 64 years in North Carolina census tracts for the years 2017 to 2019. Heart disease deaths were defined as deaths with International Classification of Disease, 10th revision (*ICD-10*) I00–I09, I11, I13, I20–I51 listed as the underlying cause. Results were age-standardized in 10-year age bands to the 2010 US Standard Population. Underlying data were made available through an agreement with the North Carolina Department of Health and Human Services, Division of Public Health.

#### RSTr: Rhode Island Hospitalization Data

To demonstrate the RSTr, we use Rhode Island hospitalization data from the Rhode Island Department of Health Hospital Discharge Data Program [34]. Specifically, we examine myocardial infarction and stroke-related inpatient hospitalizations in acute care hospitals among adults aged 20 to 69 years for Rhode Island census tracts in 2021 to 2023. Myocardial infarction and stroke hospitalizations were defined as hospitalizations with *ICD-10* I21-22, I60-63, I65-66 as the primary diagnosis. We ran an MSTCAR model for the years 2021 to 2023 by sex and 10-year age group and used the 2000 US Standard Population in 10-year age bands for age-standardization [18]. We then aggregated across sex and age-standardized across age groups. Underlying data were made available through an agreement with the Rhode Island Department of Health Hospital Discharge Data Program and are not publicly available.

### Benefit 1: Decreased Number of Geographic Units With Suppressed Data

#### Overview

**Figure 4.**
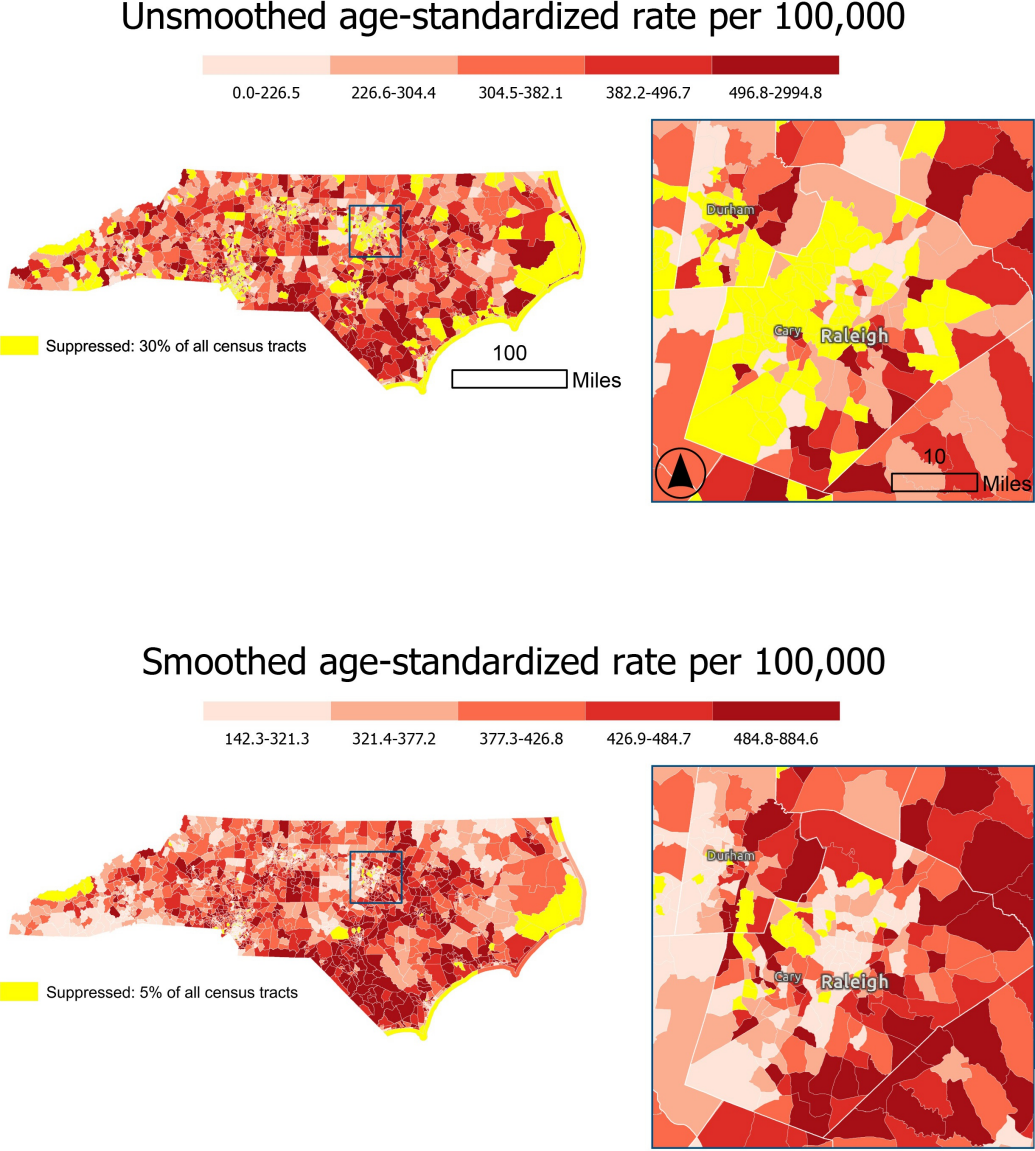
Rate Stabilizing Toolbox (RSTbx)-generated heart disease death rates by North Carolina census tract, adults aged 35 years and older, 2017‐2019. The rates displayed in the top map are unsmoothed and suppressed according to United States Cancer Statistics suppression criteria; the rates displayed in the bottom map are spatially smoothed and use suppression criteria based on relative precision using a 95% CI. Comparison of the smoothed and unsmoothed rates shows the decreased percentage of census tracts that have suppressed rates when using RSTbx, demonstrating Benefit 1. Note that cut points on the maps differ, reflecting the attenuation of variance in the spatially smoothed rates.

An important advantage of the underlying Bayesian statistical models in RSTbx and RSTr is their ability to increase the precision of estimates, thereby offering the potential to produce reliable estimates even when the event counts and/or population sizes are small. Compared to crude, unmodeled estimates, RSTbx and RSTr generate reliable estimates for a greater number of geographic units and therefore display fewer suppressed geographic units on maps (Figures4  5). In these figures, estimates are deemed reliable based on their 95% CI. Additionally, the models used by these tools attenuate outliers and therefore narrow the range of estimates after spatial smoothing. This change reflects the improved precision and reliability of estimates that are otherwise sporadically high or low.

**Figure 5.**
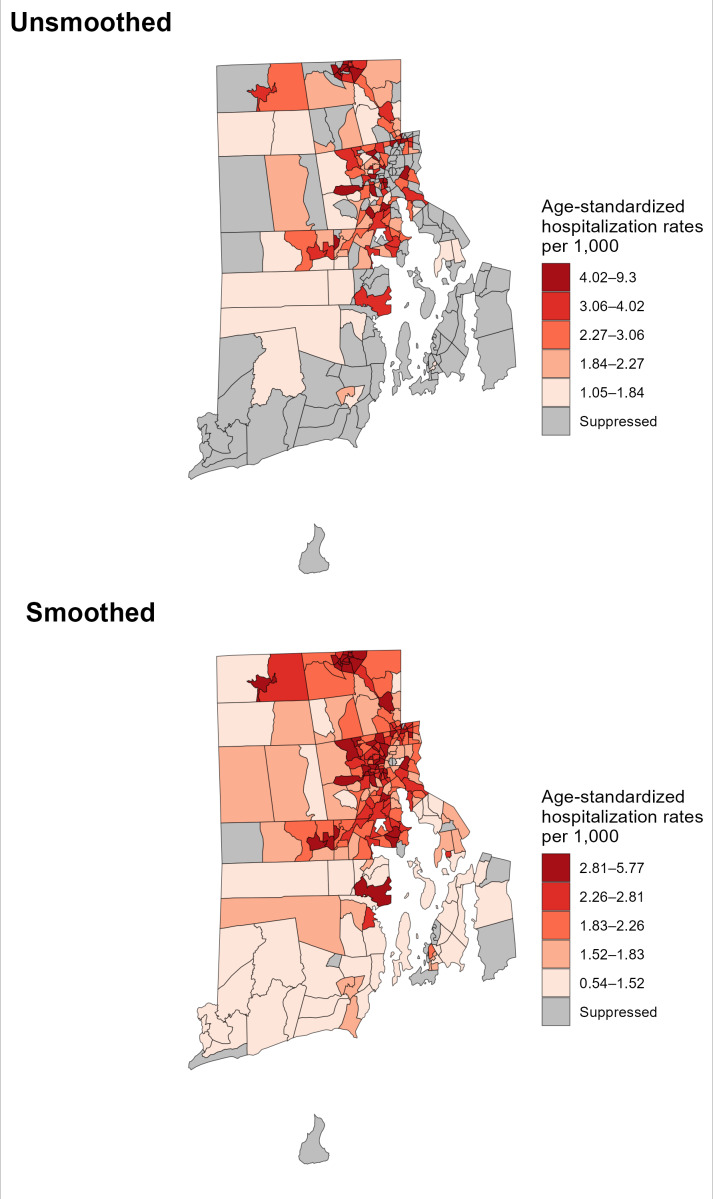
Rate Stabilizing Tool for R (RSTr)-generated myocardial infarction and stroke inpatient hospitalization rates by Rhode Island census tract, adults aged 20‐69 years, 2021‐2023. The top map displays unsmoothed hospitalization rates suppressed according to the Rhode Island Department of Health Small Numbers Policy suppression criteria; the bottom map shows spatially smoothed hospitalization rates and use suppression criteria based on relative precision using 95% CIs and an overall census tract population threshold of 100. Comparison of the smoothed and unsmoothed rates shows the decreased percentage of census tracts that have suppressed rates when using RSTr (50% and 7%, respectively), demonstrating Benefit 1. Note that cut points on the maps differ, reflecting the attenuation of variance in the spatially smoothed estimates.

#### Benefit 1: RSTbx Demonstration

Figure 4 displays age-standardized heart disease death rates for adults aged 35‐64 years by census tract in North Carolina for the years 2017‐2019. In the top map of unsmoothed death rates, census tracts with fewer than 16 deaths are suppressed according to United States Cancer Statistics guidelines [35]. Using these guidelines, 30% (n=656) of census tracts were suppressed. The suppressed census tracts were distributed across the state, particularly in coastal regions and urban areas. The bottom map of Figure 4 displays death rates that were spatially smoothed using RSTbx. Here, applying the default threshold for reliability (which is based on the 95% CI) resulted in 5% (n=105) of census tracts being suppressed.

#### Benefit 1: RSTr Demonstration

Figure 5 displays age-standardized myocardial infarction and stroke inpatient hospitalization rates for adults aged 20 to 69 years by census tract in Rhode Island for the years 2021 to 2023. In the top map, estimates are suppressed according to the Rhode Island Department of Health Small Numbers Reporting Policy [36]; the bottom map is spatially smoothed and suppressed based on precision and population. The Rhode Island Department of Health Small Numbers Policy features data suppression recommendations for a wide variety of data types and data reporting scenarios. In the case of the hospitalization data, several layers of consideration about the data are needed to determine whether or not to suppress rates. In brief, the rates suppressed in the unsmoothed map take into consideration small numerators (number of hospitalizations) paired with large denominators, leading to unreliable or unstable rates. The suppression criteria result in 50% (n=122) of census tracts being suppressed. The suppressed census tracts were primarily located in small Rhode Island towns and villages, especially small coastal towns and villages.

For the spatially smoothed map, relative precision was calculated using a 95% CI and resulted in 7% (n=17) of census tracts being suppressed with an overall census tract population threshold of 100. Additionally, the smoothed map demonstrates an extension of the spatial patterns in the unsmoothed map. The inner census tracts of Providence have more tracts in the higher categories than in the unsmoothed data, and many less dense areas have low hospitalization rates. In this example, the increase in the number of reliable estimates using the smoothed estimates tells a clearer version of the story presented by the unsmoothed data.

### Benefit 2: Flexibility to Set the Threshold for Reliability for Small Area Estimates

#### Overview

The default threshold for reliability for both RSTbx and RSTr is set at the commonly used 95%. However, RSTbx and RSTr both allow users to relax that threshold to less than 95%. Both tools use the definition in Quick and Song [13] to deem an estimate “reliable” if its relative precision (ie, the ratio of the posterior median and the width of the CI) is greater than 1. RSTbx output includes the maximum CI for which each estimate is deemed reliable, known as its level of reliability. RSTr allows users to relax the threshold for reliability by calculating the relative precision using narrower CIs (eg, 90% or 80%).

This feature allows users to more completely visualize geographic patterns due to less suppression at lower levels of reliability. This advantage, however, comes with a caveat: at some point, with a low enough threshold for reliability (eg, 70%), all geographic units would be considered to have “reliable” estimates. However, estimates with low levels of reliability may be based on very small numbers, leading to potential privacy concerns. Therefore, relaxing the reliability level may need to be accompanied by additional suppression criteria based upon population size.

#### Benefit 2: RSTbx Demonstration

**Figure 6.**
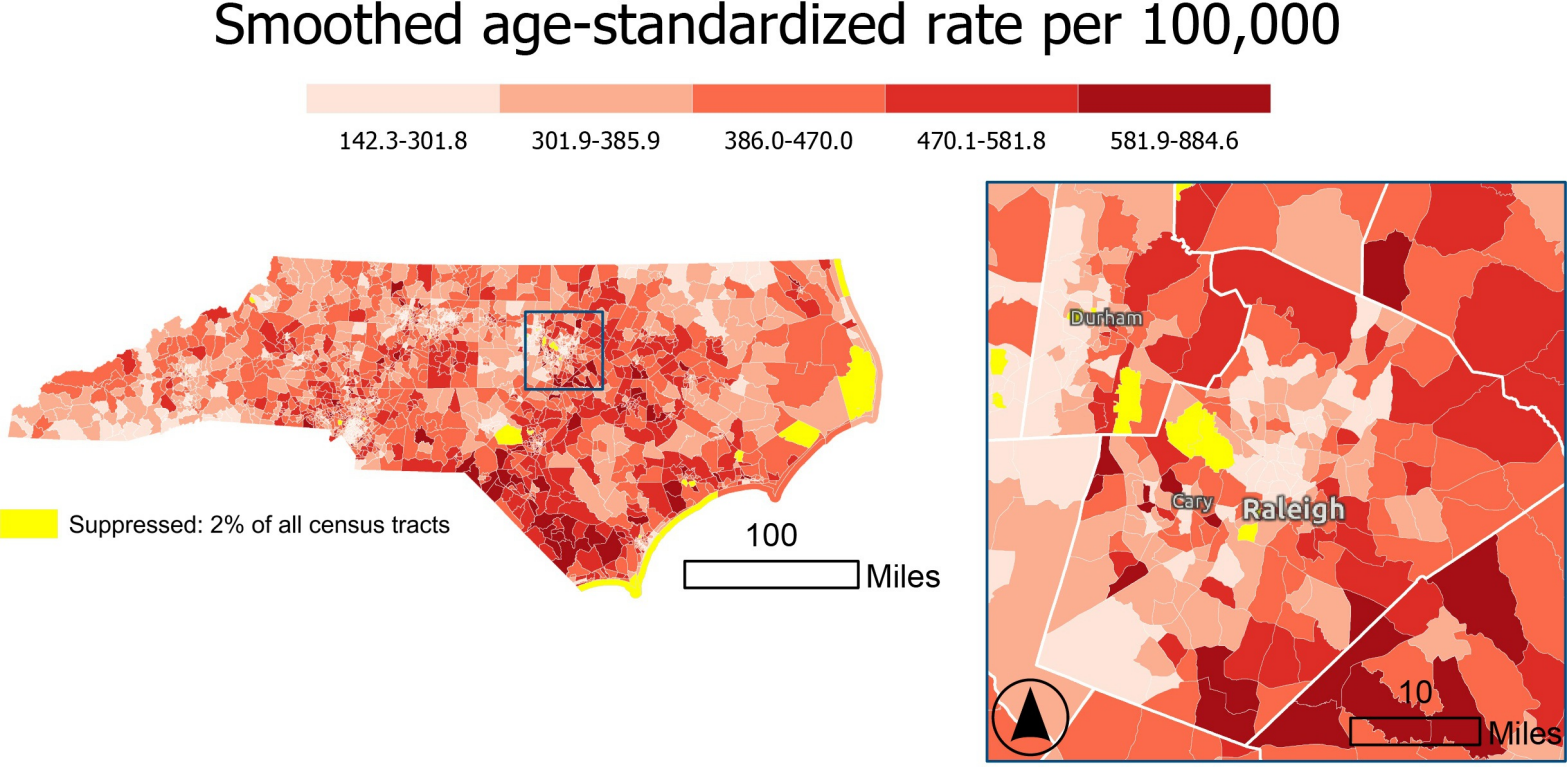
Rate Stabilizing Toolbox (RSTbx)-generated heart disease death rates by North Carolina census tract, adults aged 35 years and older, 2017‐2019. The map displays spatially smoothed rates, and reliability is defined by relative precision using 90% CIs. Comparison of this figure with Figure 4 shows the decrease in the percentage of census tracts with suppressed rates when using a relaxed precision level, demonstrating Benefit 2.

Figure 6 displays North Carolina heart disease mortality rates for census tracts that meet the threshold for reliability using the 90% CIs. Relaxing the threshold for reliability from 95% in Figure 4 to 90% decreases the percent of census tracts that are suppressed from 5% to 2%.

**Figure 7.**
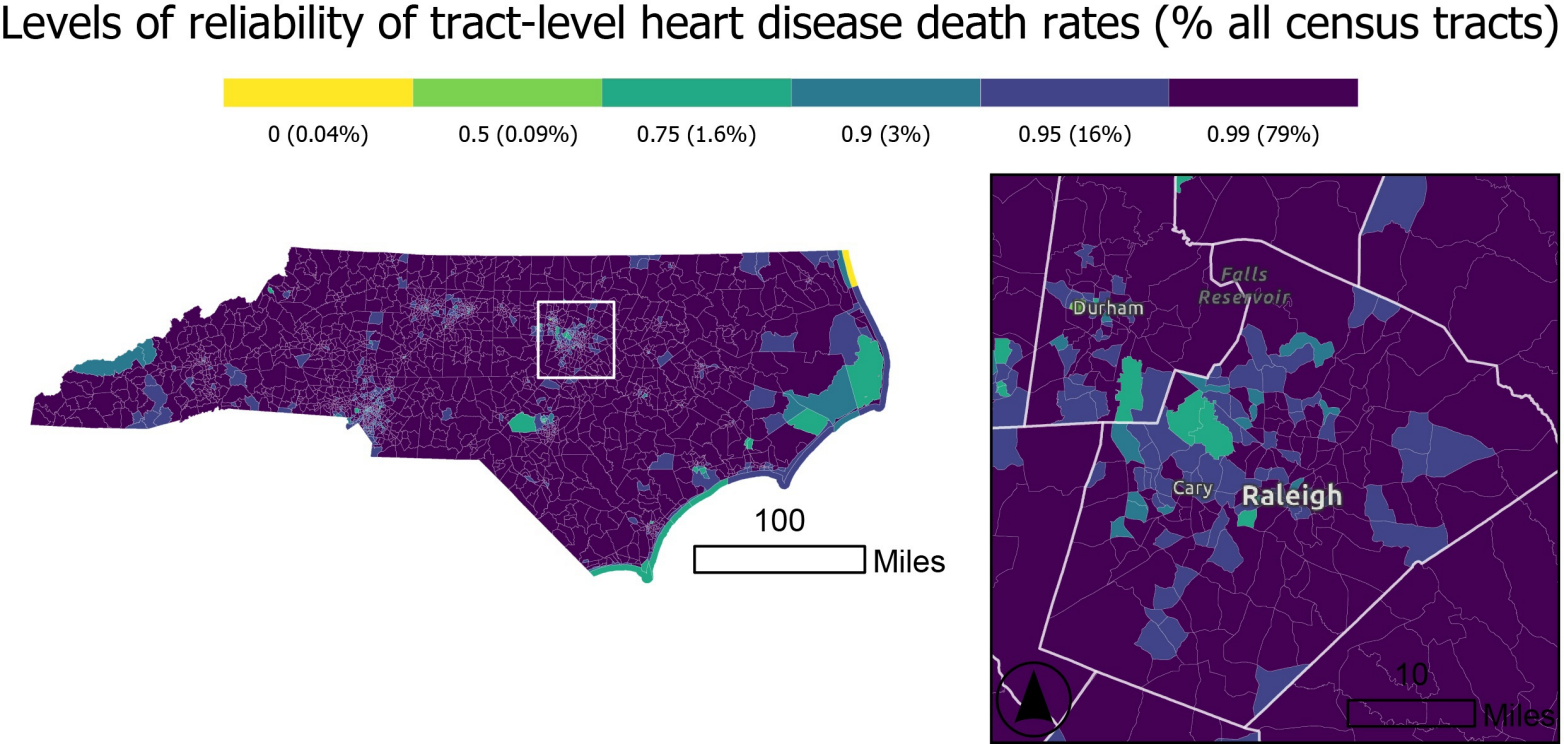
Levels of reliability of Rate Stabilizing Toolbox (RSTbx)-generated age-standardized heart disease death rates by North Carolina census tract, adults aged 35 years and older, 2017‐2019. This map shows the maximum credible interval level at which stable and reliable smoothed estimates can be produced, illustrating the spatial variation in the quality of RSTbx-derived estimates and demonstrating Benefit 2. Wider credible intervals reflect greater uncertainty, which tends to occur in tracts with smaller total population estimates and lower case counts.

RSTbx also permits users to map geographic units by their level of reliability since those are included in the RSTbx output. Figure 7 displays the level at which the estimate for each census tract is reliable. Estimates for much of the state are reliable at or above 95%. From this map, users can easily see geographic patterns in the reliability of the estimates and the range of reliability across estimates. As demonstrated in Quick and Song, these maps can be viewed as mapping a measure of uncertainty, as they highlight the geographic variation in the amount of information contributed by each geographic unit [15].

#### Benefit 2: RSTr Demonstration

**Figure 8.**
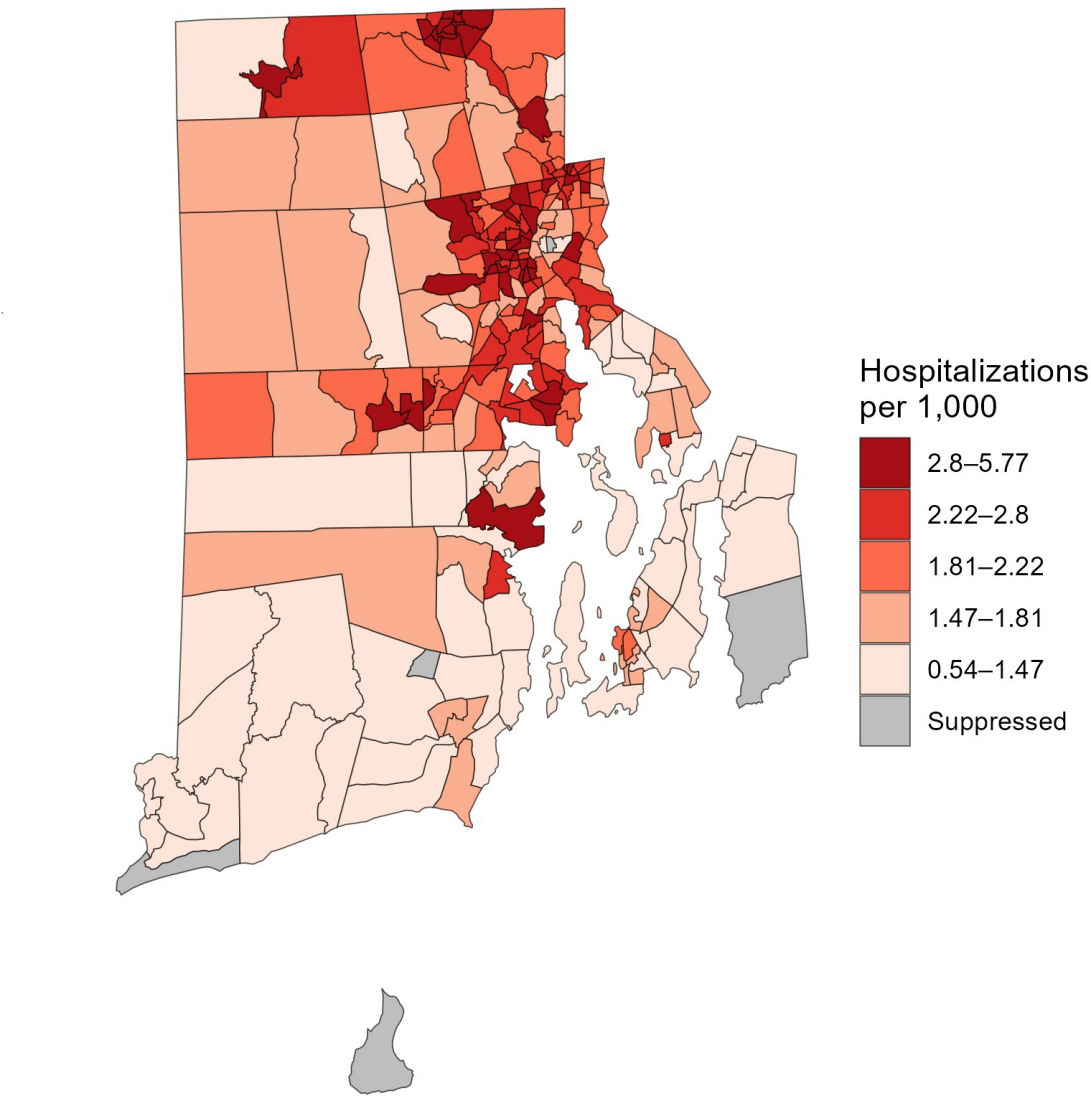
Rate Stabilizing Tool for R (RSTr)-generated myocardial infarction and stroke hospitalization rates by Rhode Island census tract, adults aged 20‐69 years, 2021‐2023. The map displays spatially smoothed rates. Reliability is defined by relative precision using a 90% CI. Comparison of this figure with Figure 5 shows the decrease in the number of census tracts with suppressed rates using a relaxed precision level, demonstrating Benefit 2. A total of 5 (2%) of census tracts are suppressed.

Figure 8 displays Rhode Island myocardial infarction and stroke in-patient hospitalization rates for census tracts that meet the reliability criteria using a 90% CI. Relaxing the CI for the reliability criteria from 95% in Figure 5 to 90% further decreases the percent of census tracts that are suppressed (from 7% to 2%), but includes less precise estimates.

Geographic patterns in the levels of reliability (ie, the maximum CI whose width is less than the posterior median) will closely align with the population sizes of the geographic units. However, unlike maps of population sizes—where a given population size may be sufficient to produce reliable estimates for a common outcome and be insufficient to produce reliable estimates for a rare outcome—maps of the reliability levels will be standardized across outcomes. That is, estimates from analyses of different outcomes in different datasets with the same level of reliability can be viewed as being equally precise on a relative basis.

### Benefit 3: Using Credible Intervals to Identify Statistically Significant Differences Between Places

#### Overview

**Figure 9.**
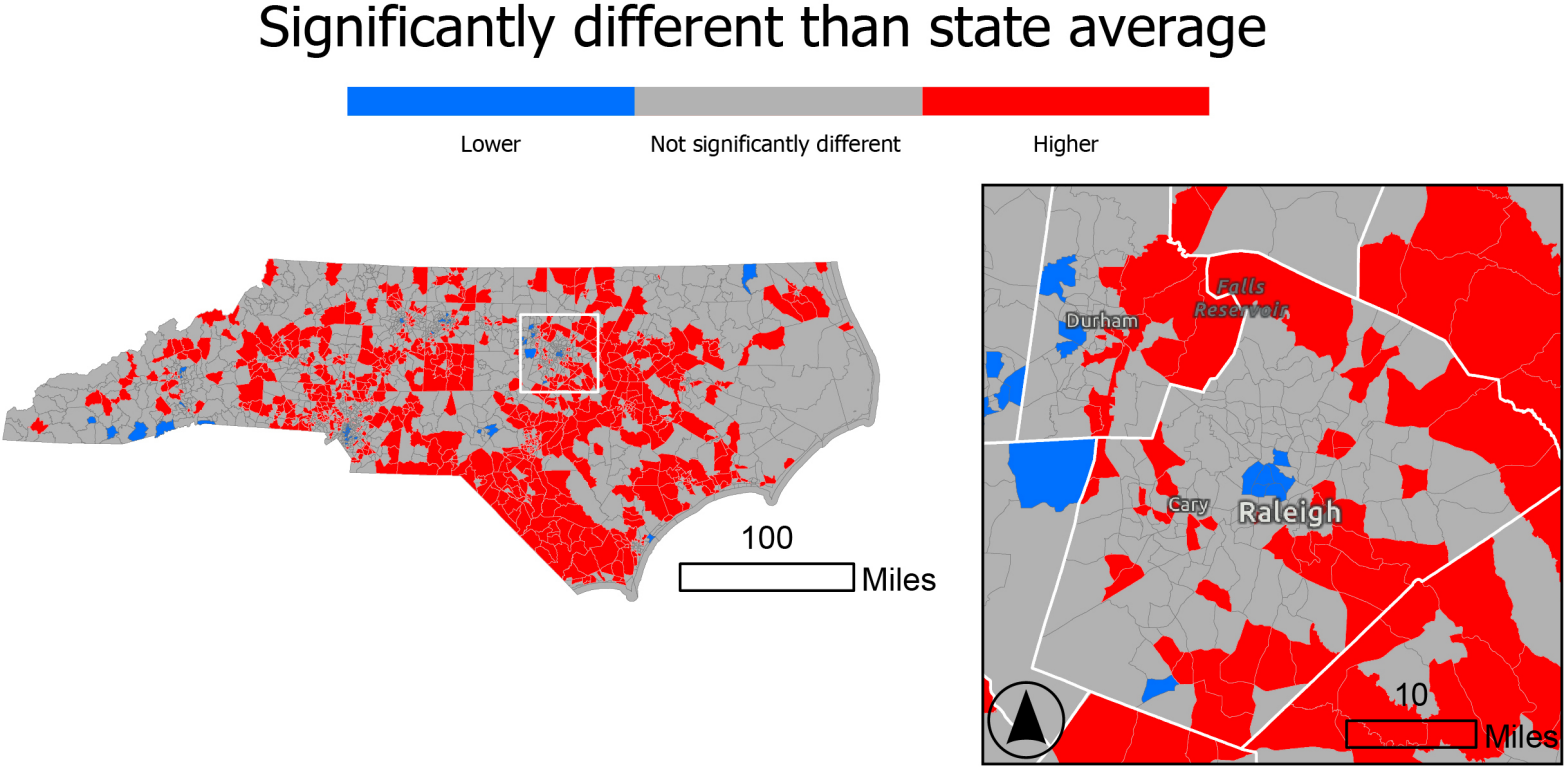
Comparison of Rate Stabilizing Toolbox (RSTbx)-generated census tract level heart disease death rates to the state rates, ages ≥35 years, 2017‐2019, North Carolina. By using 95% CIs that are generated for each census tract, this map shows census tracts with heart disease death rates that are statistically significantly higher or lower than the North Carolina heart disease death rate, demonstrating Benefit 3.

Another benefit of RSTbx and RSTr is the ability to compare estimates for geographic units to another value. This other value may be a single value (eg, a state rate) or another estimate (eg, the census tract with the lowest estimate). This comparison is made by using the CI at a specified level (eg, 95%). Estimates with a CI that excludes a value or that does not overlap with another CI are determined to be statistically different. Importantly, estimates that are unreliable can still be statistically significantly different from a comparison rate. As demonstrated in Figures9  10, this approach can be used to highlight geographic units (eg, census tracts) that are statistically higher or lower than the state rate.

**Figure 10.**
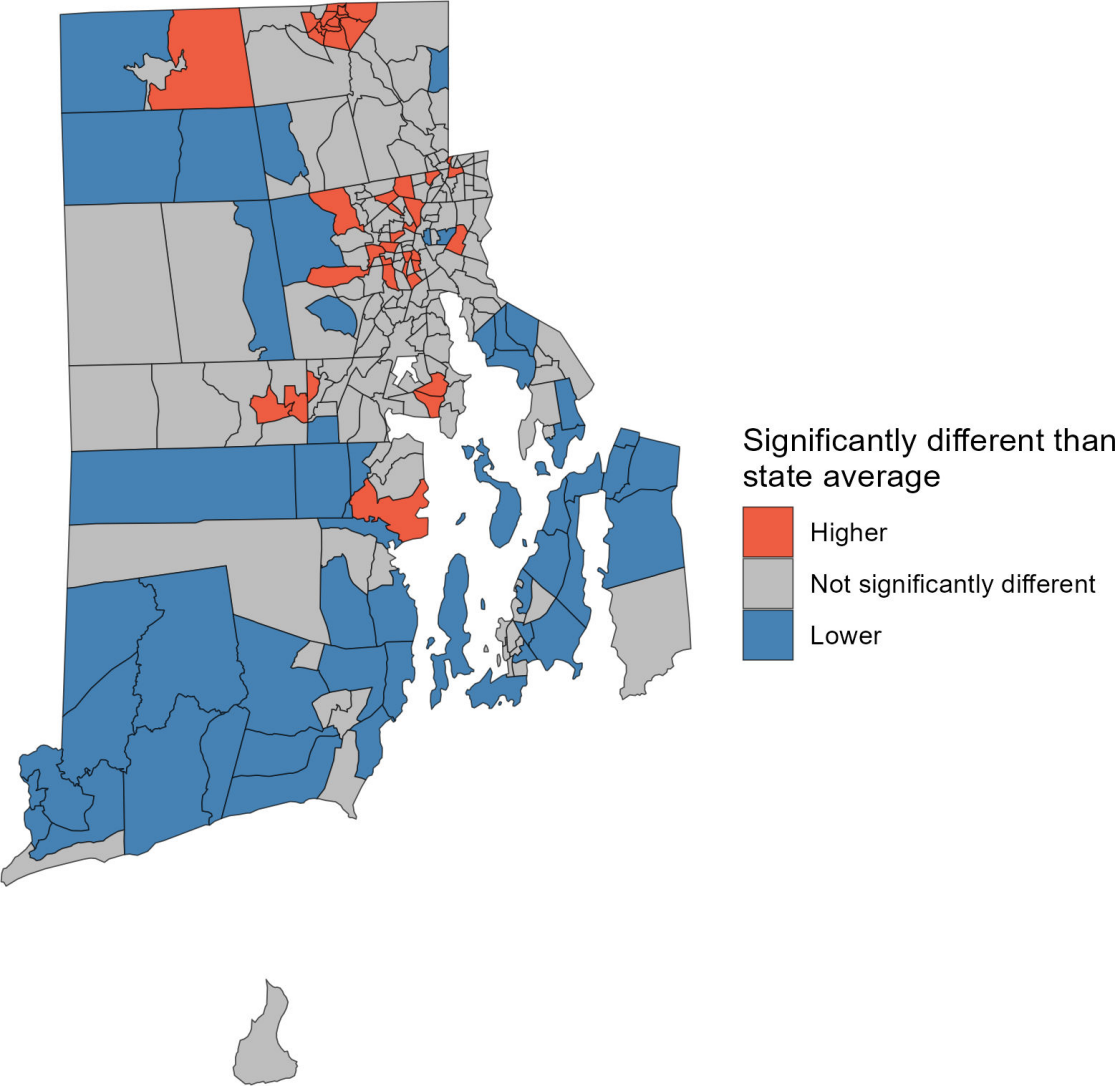
Comparison of Rate Stabilizing Tool for R (RSTr)-generated census tract level myocardial infarction and stroke hospitalization rates to the state rates aged 20‐69 years, 2021‐2023, Rhode Island. By using 95% CIs, this map shows census tracts with hospitalization rates that are statistically significantly higher or lower than the Rhode Island hospitalization rate, demonstrating Benefit 3.

#### Benefit 3: RSTbx Demonstration

Figure 9 displays census tracts in North Carolina for which the age-standardized, spatially smoothed heart disease death rates are statistically higher (red) or lower (blue) than the state level. Statistical significance is determined when the 95% CI for the tract level death rates do not include the state level value. Census tracts with rates that are statistically different from the state rate are found across the state. Notably, statistically higher rates are concentrated in the state’s urban areas.

#### Benefit 3: RSTr Demonstration

Figure 10 displays census tracts in Rhode Island for which the age-standardized, spatially smoothed myocardial infarction and stroke in-patient hospitalization rates are statistically higher (red) or lower (blue) than the state rate. Statistical significance is determined when the 95% CI for the tract level death rates do not include the state rate. Census tracts shown in gray have estimates that are not significantly different from the state hospitalization rate.

**Figure 11.**
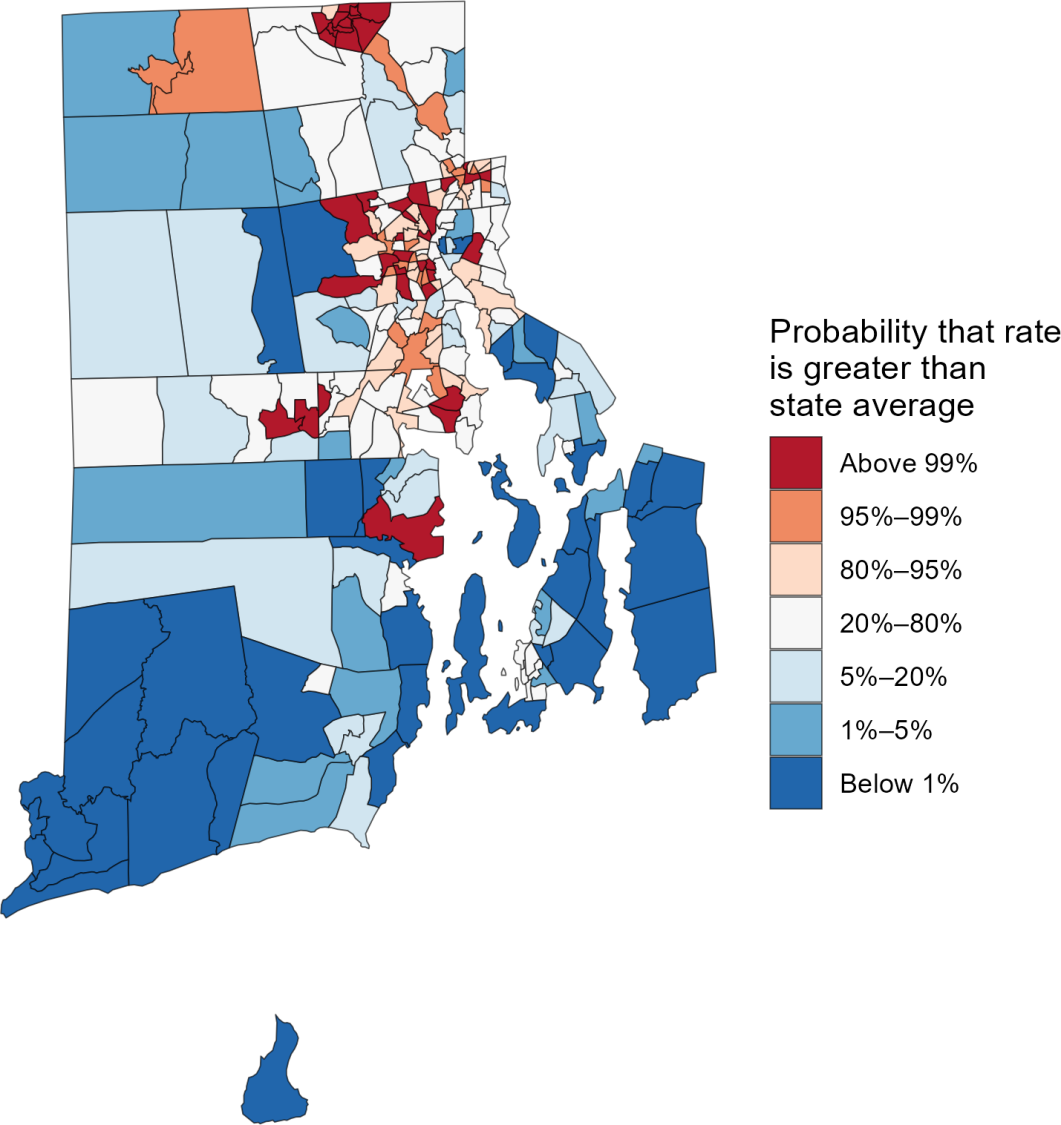
Probability that Rate Stabilizing Tool for R (RSTr)-generated myocardial infarction and stroke hospitalization rates by census tract are greater than the state value, adults aged 20‐69 years, 2021‐2023, Rhode Island. This is an extension of Benefit 3.

Additionally, the output from RSTr allows users to calculate the probability that one value is greater than another, frequently referred to as an exceedance probability. In Figure 11, we demonstrate the probability that the tract level hospitalization rates in Rhode Island exceed that of the state level. Unlike the approach in Figure 10, we are not limited to binary differences in probability, revealing a nuanced look at the statistical strength of the estimates. With RSTr’s samples, users can calculate the percent of samples for a given region, group, or time that exceed a given value. In this approach, the percent estimate implies its opposite as well: an 80% chance for an estimate to be greater than the state hospitalization rate implies a 20% chance for the estimate to be less than that rate. In Figure 11, most of the strongest probabilities of an estimate being greater than the rate for Rhode Island are clustered in urban areas, whereas rural areas tend to have the lowest probabilities of being greater than the state’s hospitalization rate, indicating that their rates may be significantly lower than the state rate.

## Discussion

### Strengths and Limitations

RSTbx and RSTr have many strengths. First, as we have demonstrated, the outputs of both RSTbx and RSTr can be used to map small area estimates or statistical comparisons, with varying thresholds for reliability. Second, these tools allow users to quickly run complex Bayesian models. Third, users can easily generate age-standardized rates. RSTbx includes integrated standardization to either the 2000 US Standard Population or the 2010 US Standard Population in 10-year age groups. RSTr users may age-standardize to any population and are able to aggregate by domains other than age, such as race, sex, year, or region.

Though software such as JAGS, Stan, and BUGS [37-39] have addressed many of the same issues as RSTr and RSTbx and provide many of the statistical models used by our tools in a more generic framework, our tools come with distinct advantages. First, RSTr features a wider family of CAR models to choose from with more complex specifications available; Stan and JAGS, for example, provide at best an MCAR model for estimation, and BUGS only provides a BYM CAR model. Additionally, since RSTbx and RSTr are designed specifically to run CAR models, their parameters and initial values are pre-specified based on existing work in the literature. More general software like Stan and BUGS requires a more hands-on approach and can lead to nonideal CAR model specification. Finally, RSTbx and RSTr utilize recent advances in CAR model methodology not seen in other statistical packages. The UCAR model implemented in both tools features enhancements that restrict the strength of the spatial smoothing using models which aim to establish a minimum on the amount of data required to yield a reliable estimate [13,15].

Both RSTbx and RSTr have limitations. First, the input data are assumed to be a census or representative sample of the population being studied. Neither tool is designed to accommodate survey weights or potential disparities in reporting quality (eg, undercounts). Any data quality issues that would cause the crude values (ie, the event counts divided by the population sizes) to be biased would also affect the quality of the estimates. Spatial data in general are prone to many limitations; nuances such as the modifiable areal unit problem, the ecological fallacy, and edge effects should all be considered when interpreting results generated by RSTbx and RSTr. Additionally, RSTbx includes only the UCAR model, meaning that smoothing occurs only over space and not over time or other domains.

Finally, our statistical models have some limitations. In contrast to the UCAR models used in both tools, RSTr’s MCAR and MSTCAR models do not feature enhancements to restrict the strength of the spatial smoothing and thus have the potential to yield estimates with relative precisions greater than one when zero events have been observed. As a result, these models may produce estimates that are overly smooth (ie, reduced geographic disparities between adjacent geographic units) and overly precise (ie, inflated levels of reliability). To address this limitation, users can flag estimates generated by RSTr as unreliable when population sizes are not sufficiently large in addition to the use of relative precision. While Quick and Song do not provide guidance regarding requirements for population sizes, others have suggested requiring population sizes be at least 30 or 100 in order to display estimates [12]. Development of restricted MCAR and MSTCAR models is an active area of research, and future updates to RSTr and RSTbx aim to include such models. Additionally, there are future plans to expand RSTbx to use open-source GIS software, such as QGIS. Future versions of RSTbx will also include imputation of censored data and further streamline the data setup process.

### Conclusion

RSTbx and RSTr facilitate the calculation of small area estimates of population health for ArcGIS and R users, respectively. Table 1 summarizes the main features for RSTbx and RSTr. Overall, both tools simplify the implementation of complicated Bayesian spatial and spatiotemporal models. Both tools also take advantage of recent methodological developments for the CAR model and easily age-standardize estimates. Using census tract-level data from North Carolina and Rhode Island, we demonstrated the benefits of using RSTbx and RSTr to generate and map small area estimates, especially when there are small numbers of events or population sizes. We focused on three key benefits of using these tools: (1) decreased number of geographic units with estimates being suppressed based on reliability criteria, (2) flexibility to set the threshold for reliability, and (3) using credible intervals to identify statistically significant differences between geographic units. In summary, RSTbx and RSTr are powerful tools that can be used to meet the demand for high-quality, local-level data to inform public health programs and tailor health promotion activities to the needs of communities across the country.

**Table 1.** Comparison of Rate Stabilizing Toolbox and Rate Stabilizing Tool for R functionality.

	RSTbx^a^	RSTr^b^
Platform	Esri^j^ ArcGIS Geoprocessing Tool(s)	R package
Geographic units	Any user-specified geographic unit^d^	Any user-specified geographic unit^d^
Census interface	The CDR^e^ is a component of RSTbx that can be used to generate age-stratified or unstratified tables using US Census decennial or American Community Survey data at the county or census tract level	No census interface; users gather any census data of interest on their own. There are R packages to assist with this, eg, tidycensus, censusapi, etc
Age-standardization	Yes	Yes
CAR^f^ models^g^	CAR	CAR, MCAR^h^, MSTCAR^i^
Input data format	Event/population data: Geodatabase table dBASE (.dbf) Delimited files Comma-delimited files (.csv, .txt, .asc) Tab-delimited files (.tsv and .tab) Pipe-delimited files (.psv) Boundary file: Geodatabase feature GeoPackage feature GeoParquet Shapefile	Event/population data: A list of arrays whose dimensions depend on the input data, including vector (UCAR), matrix (MCAR), and three-dimensional array (MSTCAR) Adjacency data: A list of vectors which represent the index of their neighboring regions
Additional tools/software needed	ArcGIS Pro	R
Output data format	The following table formats are supported as outputs: Geodatabase table dBASE (.dbf) Delimited files Comma-delimited files (.csv, .txt, and .asc) Tab-delimited files (.tsv and .tab) Pipe-delimited files (.psv) By default, tables are written to the current ArcGIS project default geodatabase	Outputs an RSTr model object from which a long table containing estimates, events, populations, CIs, and relative precisions can be extracted. The model object requires additional processing for age-standardization and suppressed estimates
Group aggregation	None	Geographic units, race/ethnicity, sex, year
Reliability calculations	Generated automatically in the output data	Generated automatically in the output data
Probability calculations	Not calculable with the output data	Not directly available, but can be generated by user

a RSTbx: Rate Stabilizing Toolbox.

b RSTr: Rate Stabilizing Tool for R.

c Esri: Environmental Systems Research Institute.

d Assumes that the user has numerator and denominator data for the same geographic unit (eg, census tract, county)

e CDR: Census Data Retriever.

f CAR: conditional autoregressive.

g For a description of the CAR models used in RSTbx and RSTr, please refer to the overview section for that specific software.

h MCAR: multivariate CAR.

i MSTCAR: multivariate spatiotemporal CAR.

## Abbreviations

ACS: American Community Survey
BYM: Besag, York, and Mollié
CAR: conditional autoregressive
CDR: Census Data Retriever
ICD-10: International Classification of Disease, 10th revision
IDP: Individual Data Processor
MCAR: multivariate CAR
MSTCAR: multivariate spatiotemporal CAR
RST: Rate Stabilizing Tool
RSTbx: Rate Stabilizing Toolbox
RSTr: Rate Stabilizing Tool for R
UCAR: univariate CAR
